# F97. CHRONIC PHYSICAL MULTIMORBIDITIES, GENDER DISPARITIES AND TREATMENT OUTCOME IN SCHIZOPHRENIA

**DOI:** 10.1093/schbul/sby017.628

**Published:** 2018-04-01

**Authors:** Igor Filipčić, Ivona Šimunović Filipčić, Ena Ivezić, Sanja Devčić, Davor Bodor, Branka Restek Petrović, Ivana Bakija, Paola Presečki, Silvana Jelavić, Nino Mimica, Katarina Matić, Dina Librenjak, Nenad Jakšić, Žarko Bajić

**Affiliations:** 1 Psychiatric Hospital Sveti Ivan; 2 University Hospital Center Zagreb

## Abstract

**Background:**

Increased physical morbidity in patients with schizophrenia (SCH) is well established. However, our knowledge on the role of gender in chronic physical multimorbidities (CPM) remains limited, and the evidence about the effect of CPM on SCH treatment outcome is sparse. The present study explored the gender-dependent differences in the prevalence, and age of onset of CPM between SCH and the general population (GEP), as well as the effect of CPM on hospital readmission in patients with SCH.

**Methods:**

This cross-sectional study was nested within the larger frame of a prospective cohort study conducted at Psychiatric Hospital ‘‘Sveti Ivan’’, Croatia. Data were collected for a consecutive sample of 136 (49 female and 87 male) patients diagnosed with SCH (ICD-10) and 861 (467 female and 394 male) participants from the general population. The primary outcome was the prevalence of CPM. A secondary outcome was the number of psychiatric readmissions since diagnosis.

**Results:**

In the total sample we observed the significant difference in CPM prevalence between SCH and GEP in the youngest age group, <35 years old (p=0.006). Among the male participants <35 years old, there were no significant differences in the prevalence of CPM between SCH (25%) and GEP (15%) (p=0.216). However, among the female participants <35 years old, the difference was significant and clinically relevant (p=0.002). Prevalence of CPM was 50% in SCH patients, and 14% in GEP. After the adjustment for age, sex, a number of psychiatric comorbidities and duration of SCH, the number of physical illness comorbidities was significantly associated with the number of previous psychiatric hospital readmission. (multivariate, robust regression; B=0.98; β=0.24; p=0.022). Approximately, the number of rehospitalizations increases for one with each chronic physical illness.

**Discussion:**

This study identified gender differences in the prevalence of CPM in SCH patients, and the significant association of CPM with psychiatric hospital readmission. Higher physical morbidity points to a substantial disadvantage of female patients early in the course of illness. Understanding the nature and biological basis of gender-determined differences in risk and outcome of CPM might help to identify new therapeutic targets, allow more individualized treatment, and facilitate better risk prediction and application of healthcare resources.

